# Circular Production, Designing, and Mechanical Testing of Polypropylene-Based Reinforced Composite Materials: Statistical Analysis for Potential Automotive and Nuclear Applications

**DOI:** 10.3390/polym15163410

**Published:** 2023-08-15

**Authors:** Abrar Hussain, Vitali Podgursky, Dmitri Goljandin, Maksim Antonov, Fjodor Sergejev, Illia Krasnou

**Affiliations:** 1 Department of Mechanical and Industrial Engineering, Tallinn University of Technology, Ehitajate Tee 5, 19086 Tallinn, Estonia; vitali.podgurski@taltech.ee (V.P.); maksim.antonov@taltech.ee (M.A.); fjodor.sergejev@taltech.ee (F.S.); 2 Department of Materials and Environmental Technology, Tallinn University of Technology, Ehitajate Tee 5, 19086 Tallinn, Estonia

**Keywords:** circularity and processing, fiber-reinforced composites and injection molding, micro characterization and surface roughness measurement, macro mechanical testing and green surface tribology, sustainability

## Abstract

The circularity of polymer waste is an emerging field of research in Europe. In the present research, the thermal, surface, mechanical, and tribological properties of polypropylene (PP)-based composite produced by injection molding were studied. The pure PP matrix was reinforced with 10, 30, and 40% wt. of pure cotton, synthetic polyester, and polyethylene terephthalate post-consumer fibers using a combination of direct extrusion and injection molding techniques. Results indicate that PP-PCPESF-10% wt. exhibits the highest value of tensile strength (29 MPa). However, the values of tensile and flexural strain were lowered with an increase in fiber content due to the presence of micro-defects. Similarly, the values of modulus of elasticity, flexural modulus, flexural strength, and impact energy were enhanced due to an increase in the amount of fiber. The PP-PCCF-40% wt. shows the highest values of flexural constant (2780 MPa) and strength (57 MPa). Additionally, the increase in fiber loadings is directly proportional to the creation of micro-defects, surface roughness, abrasive wear, coefficient of friction, and erosive wear. The lowest average absolute arithmetic surface roughness value (R_a_) of PP and PP-PCCF, 10% wt., were 0.19 µm and 0.28 µm. The lowest abrasive wear value of 3.09 × 10^−6^ mm^3^/Nm was found for pure PP. The erosive wear value (35 mm^3^/kg) of PP-PCCF 40% wt. composite material was 2 to 17 times higher than all other composite materials. Finally, the single-step analysis of variance predicts reasonable results in terms of the *p*-values of each composite material for commercial applications.

## 1. Introduction

Circularity is an emerging field for the conversion of raw materials into green and sustainable products [1,2]. Innovation and new technological development have transformed open-loop manufacturing into closed-loop manufacturing [3,4]. The processing industries are focusing on the development of sustainable products [5]. Environmental pollution [6], energy crises [7], a decrease in materials’ natural resources [8], and an increase in the population [9] have enhanced the use of recycled products [10]. Potentially, the utilization of polymer materials produces pre-consumer [11], post-consumer [12], end-waste [13], and end-of-life waste [14]. Pre- and post-consumer waste, as end streams, contained more than 95% of valuable material [15]. These types of commercial waste can be introduced for the fabrication of fiber-reinforced, sandwich, and particulate composite materials [7,16,17,18]. Traditionally, composite materials are made up of matrix and fiber phases [19]. The matrix phase fundamentally controls fiber dispersion, transferring of load, and protection from environmental impacts [20]. Epoxy resins like polyester (PES) [21], polypropylene (PP) [22], and polyethylene terephthalate (PET) [23] are commercial types of synthetic waste that show good adhesion properties, productivity, mechanical properties (flexural, tensile, tribological, impact, and hardness), and moisture resistance [24,25]. Principally, the fiber phase can enhance the quality and performance of composite materials [26]. Better hybridization with reinforcement fibers (glass, carbon, ashes, cotton, jute, kenaf, etc.), [27,28,29,30,31,32] improved the strength, toughness, and the commercial use of epoxy materials in the automotive, electronic, medical, and aerospace industries [33,34,35]. Due to unsaturation, bonding ability, plasticity, recyclability, versatility in utilization and mixing, mostly manufacturing techniques like compression molding [36], hand lay-up [37], resin transfer [38], and injection molding [39] are used for the transformation of waste materials into a product [18,40].

PP is a thermoplastic material commercially used for various applications [41,42]. Chemical stability, high mechanical strength, flexibility, toughness, and resistance to heat are salient features of PP [18,43,44,45]. Polyolefins hydrocarbon materials are the main source of its production [46]. Commercial use of these materials produces huge waste pollution in the environment [13,47]. Similarly, PET is a type of smart engineering material introduced as a replacement for glass in the packaging industry due to its excellent fracture strength and low weight [48]. The post-consumer PET (more than 1000 tons) waste is collected using curbside collections and deposit systems [49]. Bottle-to-bottle (for low contamination) and super-clean recycling (for high contaminated PET bottles) techniques have been introduced for the synthesis of post-consumer PET bottles [50,51]. PES synthetic polymer is cheaper and readily available as compared to other materials [52]. Simple (C-C) and complex (C=C) PESs present good bonding ability [53], low moisture absorption, suitable adhesiveness with natural fiber enforcement, and minimal binders for composite fabrication [54]. On the other hand, natural polymers are basically derived from plants and animals [55]. Plant polymers are made of cellulose, microfibrils, lignin, and hemicellulose [56]. Cotton [57], sisal [58], ramie [59], flax [60], hemp [61], and so on, are cheaper, commercial, and readily available materials [62]. Moisture absorption, low dimensional stability, and complexity in composition are major drawbacks of natural polymeric materials [63].

The well-established injection molding technique is mostly used commercially for the recycling of polymer wastes [64,65]. This technique enables the processing of different thermoplastics [66], thermosetting [67], and elastomers [67,68]. However, parameters like impurities in wastes [69], moisture [70], heating time [71], chemical reactions [72], and thermodynamic functions cause operational problems [73]. Poor control of these parameters lowered the engineering performance of manufactured products [74]. Extrusion techniques are mostly introduced to overcome these operational problems [2]. In extrusion, the mixed (matrix and fiber materials) are pushed through a heated cylinder [75]. The compound is squeezed and passed through various heating zones. The solid compound is converted into liquid. The die shapes the liquid compound into the desired shape [76]. PP-flax [77], PP-nut fibers [78], PP-jute [79], PP-glass fiber [80], and PP-carbon fibers [81] are some examples of manufacturing fiber-reinforced composite materials from the literature. Conventionally, the produced polymer products from virgin raw materials possess optimum physical properties [82]. PP as a matrix material also helps reinforce the fabric phase to gain interfacial interaction. The presence of interfacial interactions enhances the mechanical properties of fabricated composite materials. Circularity is the utilization of polymer waste to manufacture reasonable new commercial materials [83]. The mechanical properties of recycled composite materials are manifested lower in value due to low quality and performance of polymer waste [84]. The remelting and processing of extruded products through injection molding usually enhances the performance of polymer materials [85]. In injection molding, the compound mate is fed into a chamber through a hopper. The melting chamber mostly consists of four different zones. These zones operate at different temperatures and change compound mate into liquid [18,40]. A variety of polymers, like thermoplastics and thermosetting, can be processed for high or low volumes at low cost [86,87]. Thermoplastic materials are initially melted (first step) and forced into a mold cavity (second step) through an orifice [88]. The mold is kept cold. However, due to a polymer nature, thermosetting materials are melted and forced into a hot mold cavity through an orifice [67]. Modern innovation in molding technologies has made injection molding and extrusion techniques suitable for recycling due to excellent control of physical parameters (temperature, pressure, speed, time, etc.) and fast curing [89]. PP-sisal [90], PP-wood fibers [91], PP-hemp + glass fibers [92], and PP-nylon [93] are common examples of fabrication of composite materials. After development of composite materials, the quality and performance are inspected using thermal analysis, tensile, bend, impact, abrasive, and erosive testing for commercial applications [94,95,96,97,98]. Tensile properties ensure the withstand ability, fracture ability, stiffness, and percentage of plastic deformation [99,100]. Usually, these properties are correlated with structural applications. In parallel, bend testing explored the flexural properties of recycled composite materials under compressive conditions [101]. Maximum flexural stress, strain, and elongation of plastics [102], thermosetting [103], elastomers [104], composites, and metals are investigated for customer satisfaction [105]. The degree of surface finishing is evaluated using erosion [106], fatigue, creep testing, and surface characterization [107]. Mostly, line cracks, asperities, rough regions, pits, and grooves exist on the surface of composite materials [108]. These defects simulate material failure [109] and are mostly controlled using tribological investigations [110]. Similarly, materials characterization like scanning electron microscope analysis and surface profilometer measurements [111] provide immediate results to optimize manufacturing of polymeric materials for commercial applications. Collectively, mechanical, thermal, and surface techniques assured customer satisfaction and diversity [112].

The motive of this study is to introduce the concept of circularity in polymer industries for the fabrication of smart recycled composite materials. The post-consumer (PC) cotton fabric (CF), synthetic polyester fabric (PESF), and polyethylene terephthalate fabric (PETF) waste are used as reinforced fibers to fabricate polypropylene (PP) based PP-PCCF, PP-PCPESF, and PP-PCPETF composite materials. Traditional single-step direct extrusion in association with injection molding is introduced as a processing technique to enhance the physical and thermal properties of manufactured products. The fiber loadings (PCCF, PCPESF, and PCPESF) of 0 (pure PP as reference material), 10, 30, and 40% are reported for potential commercial structural and environmental applications. A scanning electron microscope (SEM) and optical and mechanical profilometers are utilized to characterize composite materials qualitatively and quantitatively for surface and cross-sectional defects. The quality and performance of recycled composite materials are analyzed using thermal, surface, mechanical, and tribological techniques for potential commercial applications and customer satisfaction. The statistical single-factor analysis of variance is established to manifest the effect of each property of an individual composite material for commercial application. Finally, the proposed framework of closed-loop pilot production of composite manufacturing can be started as a concept of circularity (recycling of polymers with almost negligible waste) in polymer recycling industries. 

## 2. Materials and Methods

### 2.1. Selection Criteria and Specification of Polymer Waste Materials

**Figure 1:**
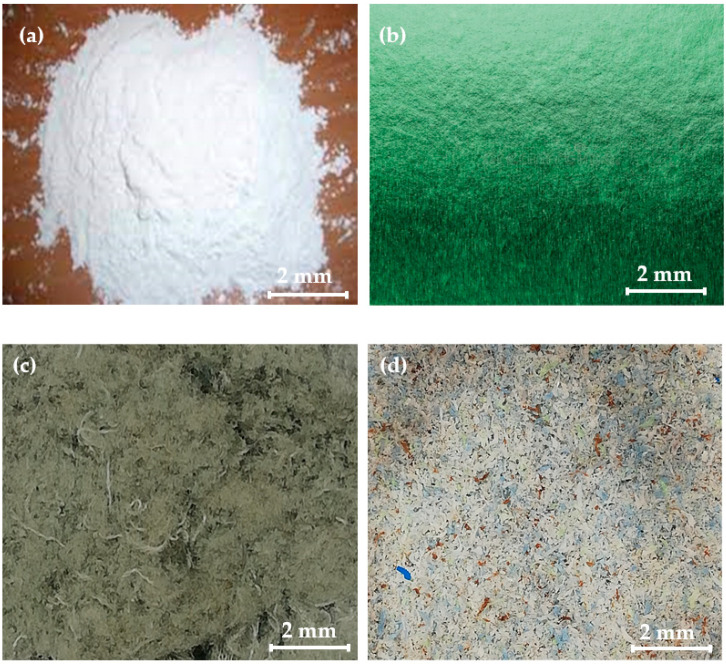
PP and ground post-consumer polymer fabric materials: (**a**) Pure PP, (**b**) PCCF, (**c**) PCPESF, and (**d**) PCPETF.

Pure polypropylene (PP) powder was used as a matrix phase. The PP powder was purchased from Egyeuroptene (Jorvas, Finland). According to the American Society for Testing and Materials (ASTM) D 1238 testing method, PP has a melt-flow index of 14 g/10 min at 2.16 kg and 210 °C at melting temperature. These properties of PP were provided by the company. The PP provides excellent adhesion, interfacial strength, moisture resistance, thermal resistance, and fiber wettability. The selected material is shown in Figure 1a.

The subjective assessment (manual), ASTM D5034-08 (Grab test) [113], and ASTM D5034-06 (Strip test) [113] are used to investigate the physical and tensile properties of PCCF, PCPESF, and PCPETF materials [113]. 

PCCF (Figure 1b) was supplied by the local industry of Estonia. The PCCF has a density of 1.55 g/cm^3^, an elongation range of 5–25% ± 1.5%, tensile strength of 0.06 MPa ± 0.001, design strength of 0.10 ± 0.004 MPa, breaking strength of 0.04 ± 0.002 MPa, and a weight of 237 g/m^2^. The PCCF was heated at 60 °C for 70 min to remove the moisture. 

PCPESF PCDT (poly-1.4 c yclohexyl-di-methylene terephthalate) (Figure 1c) was also supplied by the local industry of Estonia. The PCPESF represents post-consumer polyester fibers. The PCPES has a density of 1.45 g/cm^3^, an elongation range of 8–30% ± 1.7, tensile strength of 0.08 ± 0.004 MPa, design strength of 0.13 ± 0.004 MPa, breaking strength of 0.06 ± 0.002 MPa and a weight of 230 g/m^2^.

PCPETF (Figure 1d) was derived from post-consumer beverage bottles. The PCPETF has a density of 1.45 g/cm^3^, an elongation of 6–12 ± 2%, and a tensile strength of 90 ± 10 MPa.

Before processing, all materials were dried in an oven at 60 °C for 4 h to remove moisture and humidity.

### 2.2. Sorting, Separation, and Grinding of Post-Consumer Waste

**Figure 2:**
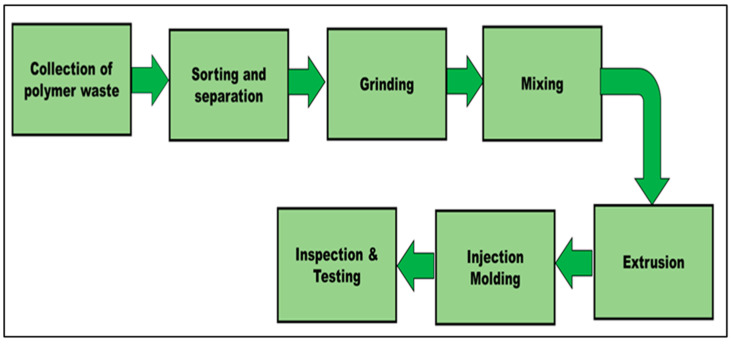
Steps of fabrication of fiber-reinforced and particulate composite materials.

After collection, the manual separation and sorting of post-consumer fabric waste (PCCF, PCPESF, and PCPETF) were performed, see Figure 2. The fabric waste in a purity range of 95–99.99% was selected for further processing. Before grinding, the fabric waste is cut into small pieces. The direct grinding of these small pieces was performed to transform fabric waste into fine fibers (PCCF and PCPESF) and flakes (PCPETF) at a speed and time of 300 rpm and 10 min, respectively [114]. The step of grinding was repeated four times to get a uniform fine-sized distribution of ground fine fibers in terms of length, diameter, and area. The SEM was used to calculate the length, diameter, and area of waste fibers. A bunch of 50 fibers (individually each PCCF, PCPESF, and PCPETF) were selected and coated with gold (Au) using a physical vapor deposition technique. A gold thin film of 2 nm was deposited, and the waste fibers were characterized. The average size distribution of waste fibers (Table 1) was measured using SEM.

### 2.3. Fabrication of Fiber-Reinforced and Particulate Composite Materials

At the start, the PP was mixed using a locally manufactured semiauto cylindrical mixer with 10, 30, and 40 wt.% fiber (PCCF, PCPESF, and PCPETF) loadings before compounding. The time and speed of mixing were 15 min and 80 rpm, respectively. In the second step, mixed polymer materials (matrix and fibers) were compounded using a twin-screw compounder Brabender extrusion machine (PLE 651-plasic corder) at temperature, speed, torque, and time of 190 °C, 40 rpm, 60 Nm, and 7 min, respectively. The extruder was operated at a temperature of 175 °C in the first zone, 180 °C in the second zone, and 190 °C in the third and fourth temperature zones. The extruded mate was shaped into long cylindrical wires.

**Figure 3:**
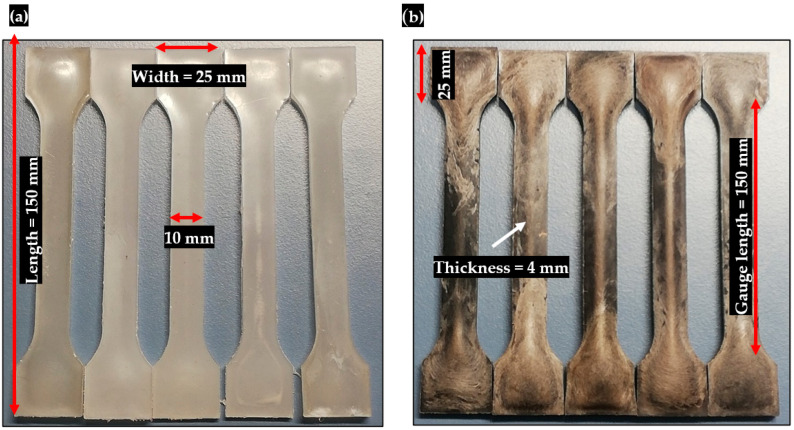
The representative images of fabricated composite materials: (**a**) Pure PP and (**b**) PP-PCCF fiber-reinforced composite materials.

The fabrication steps are shown in Figure 2. These wires were ground into pellets or beads of size 2 mm and used as raw material for injection molding. The pellets were dried for 3 h at a temperature of 60 °C. The injection molding machine (Battenfeld BA 230A) can operate at different temperature zones. Therefore, the pellets were passed through different temperature zones (120, 150, 180, and 190 °C) for melting. Finally, small pellets were injected into the mold cavity and heated at a temperature of 190 °C. The injection, cooling, and molding open times were 8 s, 25 s, and 30 s, respectively. All composite materials were designed into ASTM test specimens of size 150 mm (length) × 25 mm (width) × 4 mm (thick) products for mechanical, abrasion, surface, and erosion testing, see Figure 3a,b.

### 2.4. Quality and Performance Evaluations of Recycled Composite Materials

**Figure 4:**
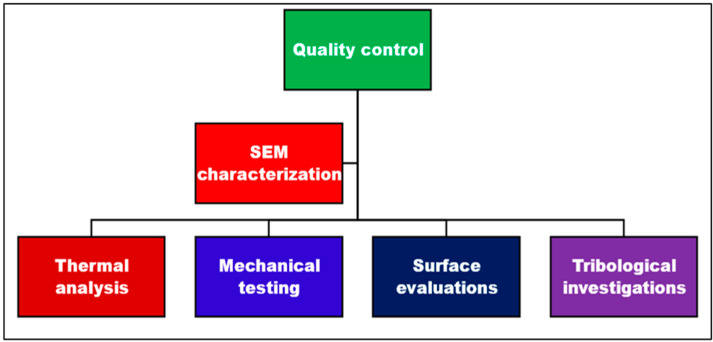
Industrial quality control and testing of materials.

The quality and performance of recycled composite materials were evaluated using thermal analysis, mechanical testing, tribological investigations, surface testing, and SEM characterization. The inspection and testing of composite materials were performed for customer satisfaction and commercial applications. The quality control diagram for the testing of materials is shown in Figure 4.

#### 2.4.1. Thermal Evaluations

**Figure 5:**
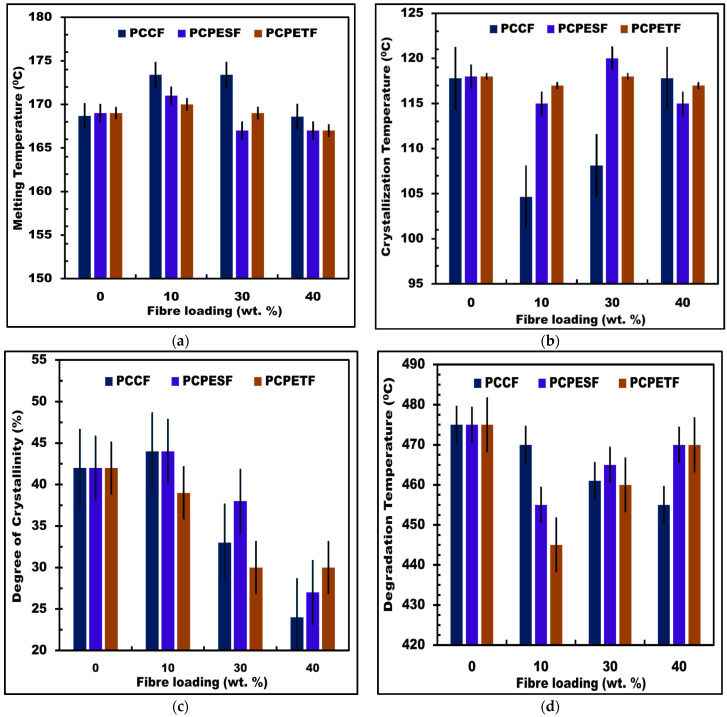
Results of DSC and TGA analysis of PP-PCESF and PP-PCPETF fiber-reinforced and particulate composite materials: (**a**) melting temperature, (**b**) crystallization temperature, (**c**) degree of crystallinity, and (**d**) degradation temperature.

The thermal differential scanning calorimetric (DSC) tests were performed using simultaneous thermal analyzers (Model STA 449 F3 Jupiter, NETZSCH Co., Houston, TX, USA). In the DSC thermal test, 10 mg of each individual composite material was heated and cooled from 0 °C to 250 °C and 250 °C to 0, respectively. The heating and cooling rate was kept constant at 15 °C/min. Moreover, pure nitrogen was used as a medium for testing at a rate of 50 mL/min. The melting temperature, degree of crystallinity, and crystallization temperature of developed composite materials were measured during changes in endothermic fusion and exothermic cooling curves. The DSC results are shown in Figure 5a–c.

Similarly, the thermogravimetric (TGA) tests were carried out using a thermogravimetric analyzer (TGA 1000 system, Anderson Materials Evaluation, Inc., Columbia, MD, USA). In the TGA thermal test, 10 mg of each individual composite material was heated from 0 °C to 600 °C at a heating rate of 10 °C/min in alumina ceramic crucibles. The balance and sample purges of pure nitrogen at the rates of 20 mL/min and 50 mL/min were used as a medium of testing. In this study, the degradation temperature of recycled composite materials was observed. The average values of each individual composite material were measured to quantify the thermal degradation for practical applications. The TGA results are depicted in Figure 5d.

#### 2.4.2. Mechanical Testing

The tensile and bending properties of manufactured composites were observed using the Universal Testing Machine (UTM Model 5820, Instron Co., Norwood, MA, USA). The tensile and flexural tests were carried out according to ASTM D3039 [115] and ASTM D5467 [116] standards at a rate of 50 mm/min. The specimens of size 4 (thickness) × 25.4 (width) × 150 mm (length) were manufactured using injection molding. The machine comprises the lower (fixed) and upper jaws (moveable). The distance between the two jaws was kept equal to the gauge length (100 mm) of the specimen. The length under analysis is known as gauge length. The gauge length of bending and tensile tests was 100 mm. The data were recorded and analyzed using Acquisition software. At least twenty tests were performed for each individual composite material. Moreover, ASTM A370 impact tests were performed to measure the impact energy of composite materials. The quantitative and qualitative analysis in terms of tensile strength, modulus of elasticity, plastic deformation, flexural strength, flexural constant, flexural strain, and impact toughness help in the prediction of performance and quality of recycled composite materials. The results are presented in Table 3 and Figures 11–13.

#### 2.4.3. Tribological Testing

The CETR Bruker UMT-2 tribometer was used to calculate the abrasive wear of developed composites. The SiC (P150 grade) sandpaper was used for the abrasion of samples. The composite pins of 4 (thickness) × 5 (width) × 25 mm (length) were slid at 0.1 m/s speed and 1 N force as a counter body on SiC sandpaper. The abrasive wear was measured for 18 m of sliding distance. The wear rate (***W***) was calculated using the following equation:

(1) $$W=\frac{V}{L\times S}$$

where ***V*** is the volumetric wear loss (mm^3^), ***L*** is the normal load (N) applied during the test, and ***S*** is the total sliding distance (m). 

The COF graph and average COF value were calculated by CETR/Bruker UMT Viewer software (https://www.bruker.com/en/products-and-solutions/test-and-measurement/tribometers-and-mechanical-testers/umt-tribolab.html, accessed on 2 July 2023). The results are shown in Figure 14 and Table 4.

#### 2.4.4. Erosion Testing

The locally manufactured four-channel accelerator erosion machine was used for determining the erosive wear of manufactured composite materials. Silica sand (SiO_2_) of 6 kg in quantity was used as an erosive medium. The size of silica sand particles was in the range of 0.1–0.6 mm. The erosion tests were repeated three times to measure the weight loss of composite materials before and after each test. The weight loss was measured using a Mettler Toledo ME204 balance with an accuracy of 0.10 mg. Moreover, impact angle, velocity, and time during the tests were 30°, 30 m/s, and 30 min, respectively. The temperature was kept at 25 °C. The results are shown in Figure 15 and Table 4. The specific weight loss M (mg/kg) at each step can be calculated using the following formula:

(2) $$M=\frac{∆m}{G\times v}$$

where ∆m, ***G***, and ***v*** are the weight loss of each sample, weight of sand, and share of sand per sample.

After the determination of ***M***, erosive wear (volumetric loss) ***E*** can be formulated as:

(3) $$E=\frac{M}{\rho }$$

where ρ is the density (mg/mm^3^) of the sample.

#### 2.4.5. Surface Characterization

The surface morphology of composite materials before and after testing was investigated by the scanning electron microscope (SEM) (Zeiss EVO^®^ MA-15 system, Oberkochen, Germany) with LaB6 cathode in the secondary electron mode, applying an accelerating voltage of 10–15 kV at a 6.5–8.5 mm working distance. About 20 fibers were selected and coated with gold using physical vapor deposition. The thickness of the gold coating was 2 nm. The fibers were characterized using SEM. The length, diameter, and area were measured digitally using specially installed software. The calculated values are shown in Table 1. 

Additionally, a mechanical profilometer (Mahr Perthometer PGK120) and an optical profilometer (Contour GT-K0+ 3D) were used to measure the surface roughness of recycled composites. The average surface roughness R_a_ (µm), root mean square roughness R_q_ (µm), maximum profile peak height R_p_ (µm), average maximum height of the profile R_z_ (µm), and maximum height of the profile R_t_ (µm) were measured and correlated with SEM micrographs. The results are shown in Figures 7–10.

## 3. Results

### 3.1. DSC and TGA Thermal Analysis of Composites

Figure 5a–d and Table 2 express the results of the DSC and TGA investigations. The composite materials produced after injection molding were under consideration. The melting, crystallization, degradation temperature, and crystallinity of pure PP were 169 °C, 119 °C, 480 °C, and 42%, respectively. Pure polymers always show sharpness in values of physical parameters (like temperature, force, stress, energy, etc.) during the investigation due to the highest purity [117].

However, fiber addition acts as an impurity. Therefore, PP-PCCF, PP-PCPESF, and PP-PCPETF (with 10, 30, and 40% fiber loadings) composite materials melt, crystallize, and degrade within a specific range of temperature [118]. The nature of fiber, length, diameter, area, density, weight, and amount of fiber affect the thermal, tensile, bending, impact [119], abrasive, and erosion properties of composite materials [120]. The degree of crystallinity of fiber-reinforced and particulate composites is calculated by using the following formula:

(4) $$\%Xc=\frac{∆H_{f}}{∆H_{f}^{o}}\times \frac{100}{w}$$

where *Xc*, *w*, $∆H_{f}$, and $∆H_{f}^{o}$ are the amount of fraction, weight, the heat of fusion of the composite material, and the heat of fusion of a 100% reference material, respectively. 

**Figure 6:**
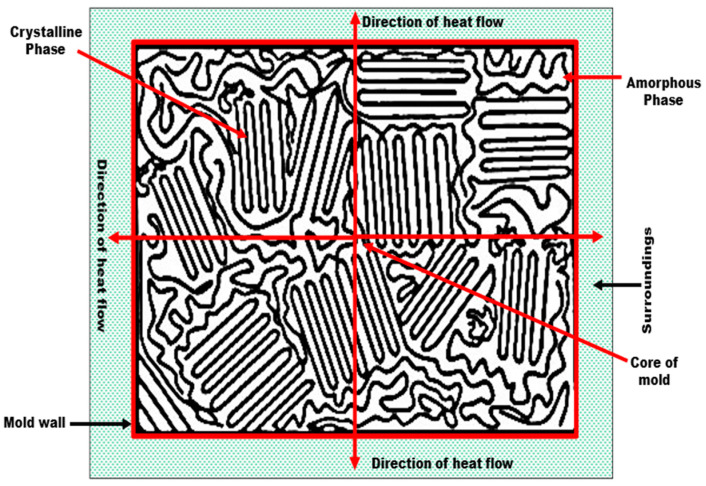
Solidification mechanism of pure PP and all recycled composite materials.

The crystallization temperature of PP-PCPESF and PP-PCPETF composites with 10 and 40% fiber amounts was constant with a value of 115 °C. The constant value of crystallization temperature appeared due to the presence of the matrix phase in higher quantities [121,122]. However, the PP-PCCF, PP-PCPESF, and PP-PCPETF composites with 30% fiber addition exhibit complex behavior due to the presence of different phases (amorphous, crystalline, and semi-crystalline), see Figure 5b and Figure 6. Similarly, the degree of crystallinity was demonstrated in Figure 5c. The level of crystallinity decreases with an increase in fiber content. Fiber nature, length, and random orientation produced amorphous and crystalline phases [123]. At the lower addition of fibers, nucleation sites come into existence. These sites increase the level of crystallinity (Figure 6). Mechanical properties fundamentally rely on the extent of crystallinity (orientation of crystals in a specific direction) [124]. Therefore, pure PP, PP-PCCF, PP-PCPESF, and PP-PCPETF composites with 10% wt. impart good mechanical properties; see Figures 11–13 and Table 3. However, a decrease in crystallinity produces brittleness, especially in manufactured composites between 30 and 40% wt. PCCF, PCPESF, and PCPETF loadings; see Figure 5c. PP-PCCF, PP-PCPESF, and PP-PCPETF composites (with 40% wt. fiber loadings) expressed the lowest crystallinity and highest brittleness; see Figures 11–13 and Table 3. An increase in crystallinity with the enhancement of 10% wt. of fiber-reinforced materials was observed. However, an increase in fiber content produces micro defects. These defects decreased the adhesion between the matrix–fiber interface and interstitial sites. Moreover, the random orientation of fibers, the nature of reinforced materials, deformation of the surface of fibers due to periodic grinding, and retention in cooling rates also caused the decrease in crystallinity of recycled composite materials between 30 and 40% wt. of fiber loadings (PCCF, PCPESF, and PCPETF). The thermal capacity and withstanding ability of produced composites are shown in Figure 5d. The degradation temperature of pure PP was 475 °C. The addition of PCCF, PCPESF, and PCPETF caused the lowering of degradation temperature. The PP-PCCF group of composite materials manifested degradability variations from 452 °C to 475 °C. The PP-PCPESF composite family possesses thermal withstand ability in the range from 455 °C to 470 °C. On the other hand, all types of PP-PCPETF composites expressed thermal capacity in the range from 445 to 470 °C. Closeness in the values of degradation temperature is due to the presence of a major PP matrix phase, matrix (PP)-fiber (PCCF, PCPESF, and PCPETF) interactions, and difference in C-C bonding [125].

### 3.2. Solidification of Composites 

Figure 6 represents the cooling model (solidification) of recycled composite materials. At a melting point of 190 °C, the melting compound mate exists in liquid form. 

**Figure 7:**
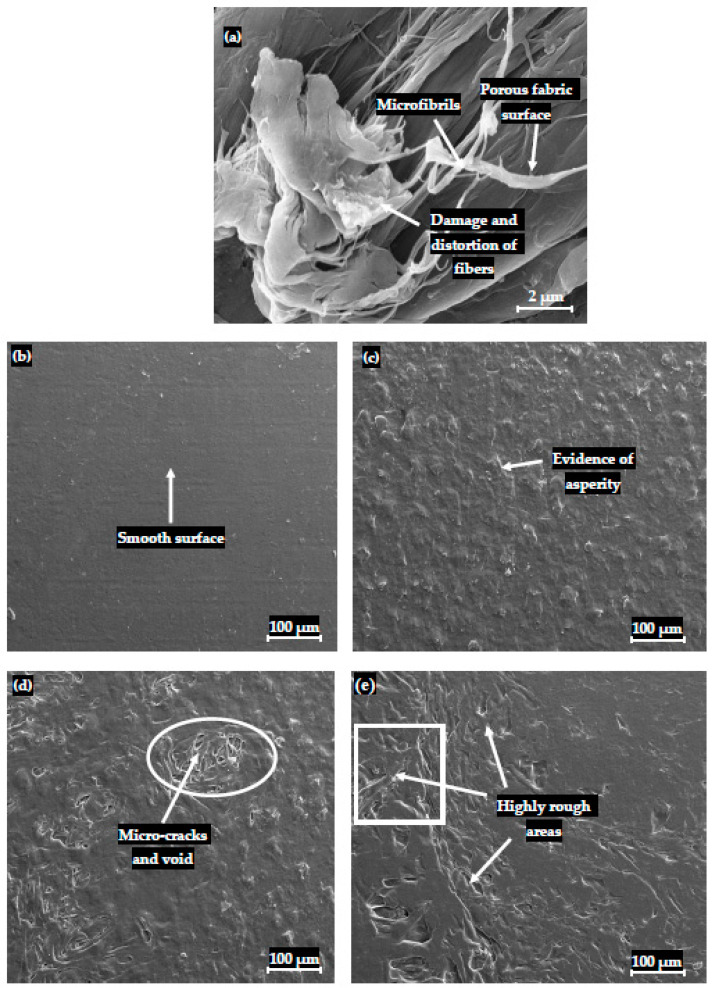
SEM analysis and surface characterization of pure cotton, PP, and PP-PCCF composites materials: (**a**) PCCF, (**b**) pure PP, (**c**) PP-PCCF 10% wt., (**d**) PP-PCCF 30% wt., and (**e**) PP-PCCF 40% wt.

**Figure 8:**
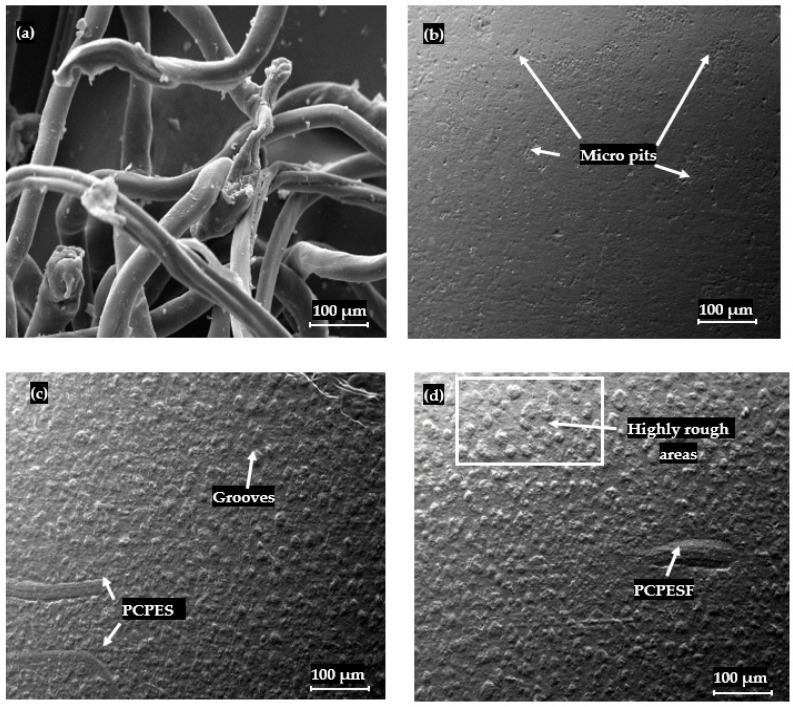
SEM analysis and surface characterization of synthetic polyester fibers and PP-PCPESF composites materials: (**a**) PCPESF, (**b**) PP-PCPESF 10% wt., (**c**) PP-PCPESF 30% wt., and (**d**) PP-PCPESF 40% wt.

**Figure 9:**
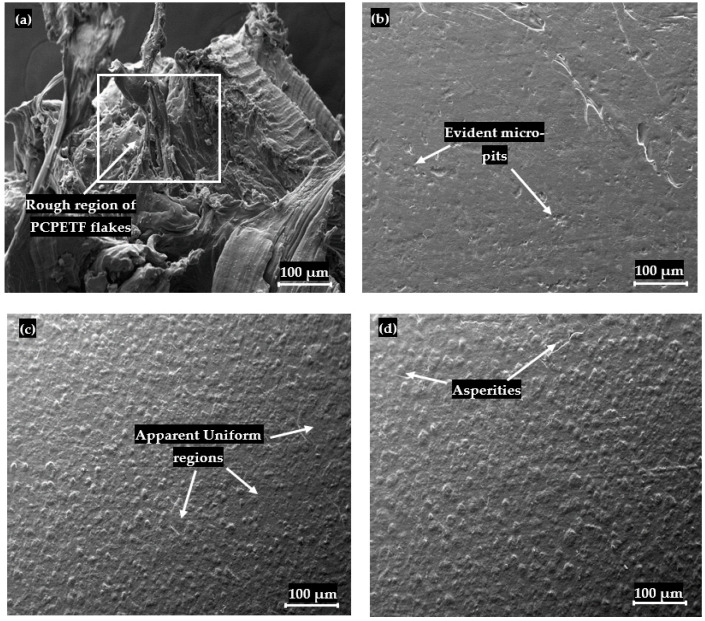
SEM analysis and surface characterization of pure polyethylene terephthalate fibers and PP-PCPETF composites materials: (**a**) PCPETF, (**b**) PP-PCPETF 10% wt., (**c**) PP-PCPETF 30% wt., and (**d**) PP-PCPETF 40% wt.

**Figure 10:**
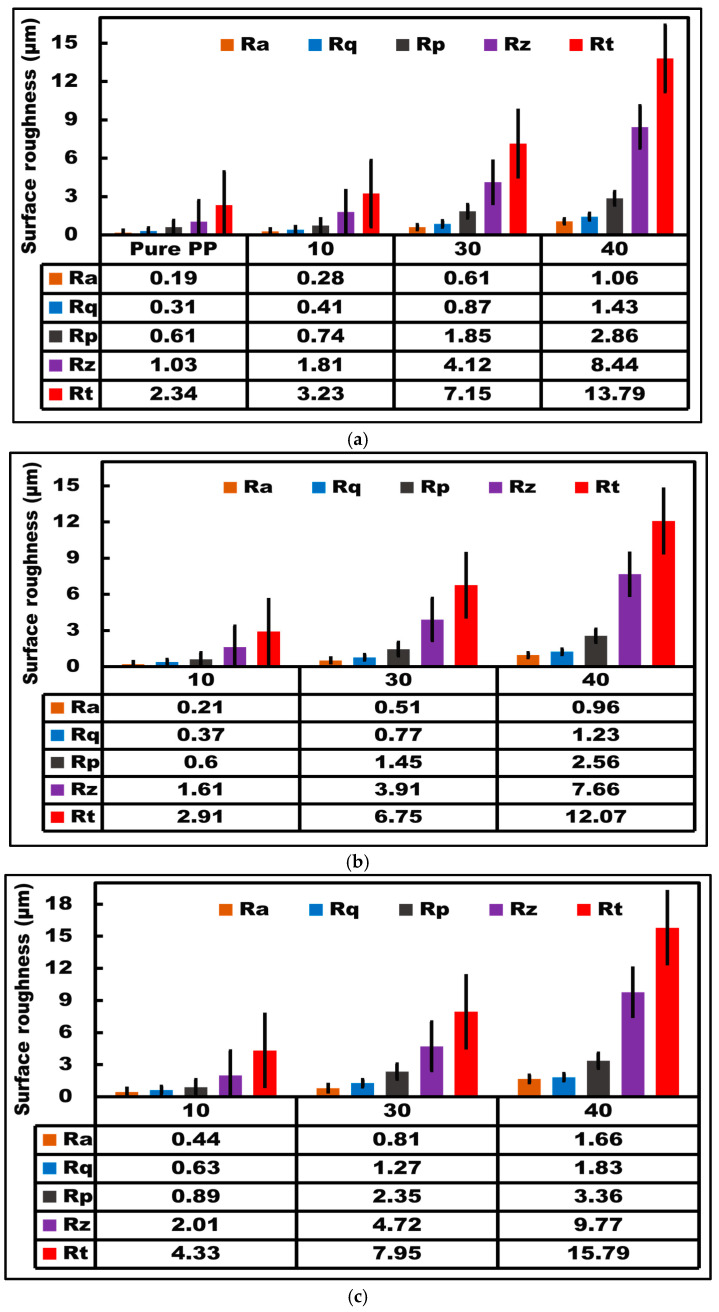
Quantitative surface analysis and average roughness parameters of developed composite materials: (**a**) PP-PCCF composite materials, (**b**) PP-PCPESF composite materials, and (**c**) PP-PCPETF composite materials.

**Figure 11:**
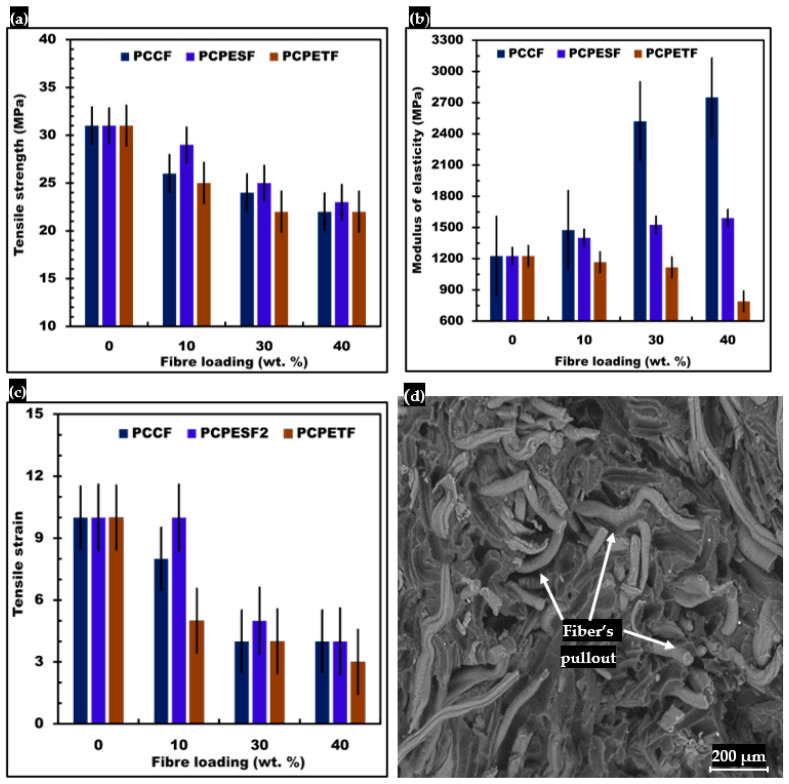
Comparative results of tensile properties of developed composite materials: (**a**) Tensile strength, (**b**) modulus of elasticity, (**c**) tensile strain, and (**d**) SEM image of fibers pull out of PP-PCPESF 40% wt. composite material after tensile test and fracture.

**Figure 12:**
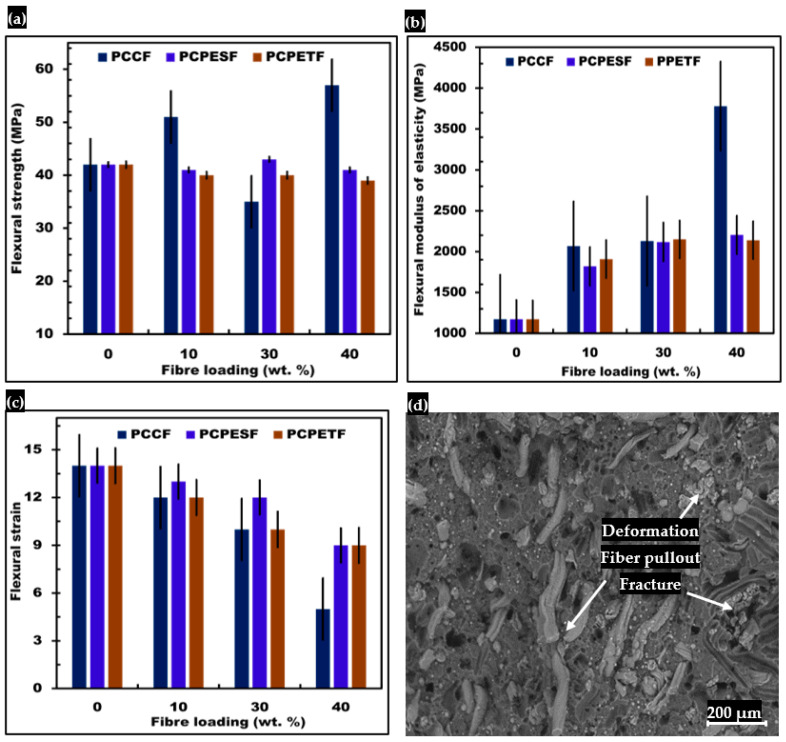
Comparative results of flexural properties of developed composite materials: (**a**) flexural strength, (**b**) flexural constant, (**c**) flexural strain, and (**d**) SEM image of fractured PP-PCPESF 40% wt. composite material after the flexural test.

**Figure 13:**
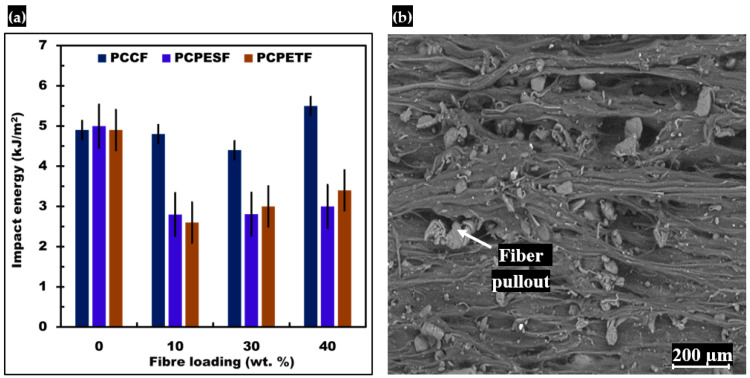
Comparative results of impact energy of developed composite materials: (**a**) impact energy, (**b**) SEM image of deformed PP-PCPESF 40% wt. composite material after impact test.

**Figure 14:**
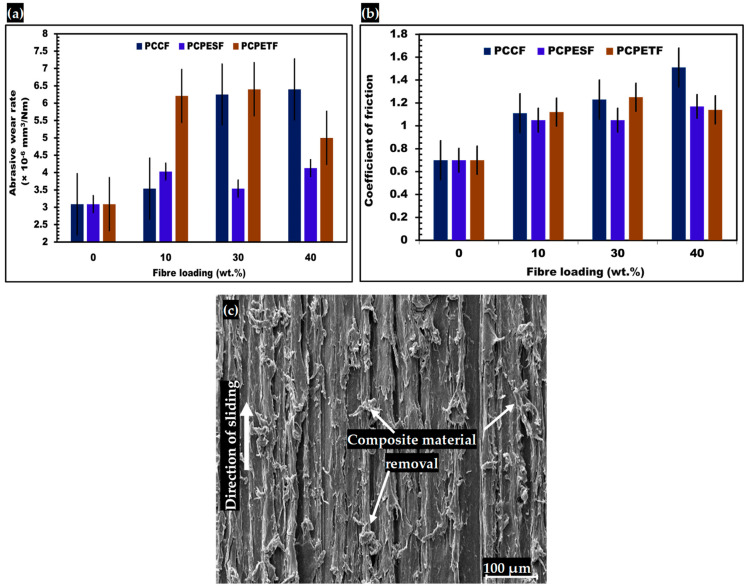
Results of abrasive wear, COF, and SEM characterization of manufactured composite materials: (**a**) results of abrasive wear rate values of PP, PP-PCCF, PP-PCPESF, and PP-PCPETF developed composite materials, (**b**) results of COF values of PP, PP-PCCF, PP-PCPESF, and PP-PCPETF fabricated composite materials, and (**c**) representative SEM image of pure PP after abrasion test.

**Figure 15:**
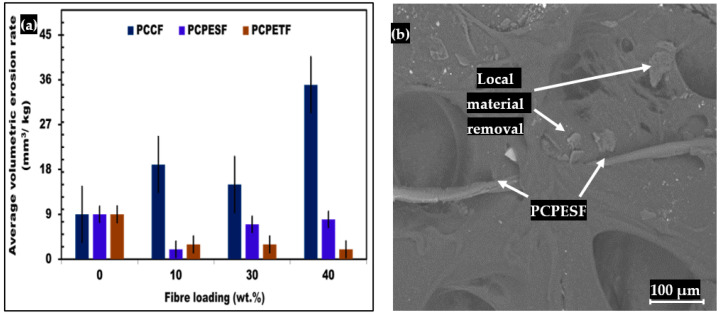
Results erosive wear and SEM characterization of recycled composites: (**a**) erosive wear rates of PP, PP-PCCF, PP-PCPESF, and PP-PCPETF developed composite materials, and (**b**) representative SEM image of PP-PCPESF-40% wt. composite material.

Thermodynamically, the heat in the form of energy flows from the core of the mold to its surroundings. Due to the flow of energy, the molecules of polymer materials start to solidify. The fibers (PCCF, PCPESF, and PCPETF) provide micro-sites to molecules of PP for solidification. Therefore, matrix–fiber interface and crystalline phases came into existence. According to Figure 6, a mostly amorphous phase exists at the boundary of the mold wall and surroundings due to heat retention. The retention of heat energy kept the temperature at a higher value at the melt mate-mold wall junction. The access to heat energy is produced by thermal barriers and heat gradients. These conditions prolonged the crystallization time of composite materials. Moreover, thermal barriers and heat gradients cause re-melting of the embryo, displacement of molecules, and random placement of reinforced (PCCF and PCPESF) and particulate fibers (PCPETF flakes). Therefore, the amorphous phase comes into existence with various surface defects, see Figure 7, Figure 8, Figure 9 and Figure 10. These micro defects play an important role in the decreasing mechanical properties of recycled composite materials (Figure 11, Figure 12, Figure 13, Figure 14 and Figure 15 and Table 3). However, the crystallization of all polymeric composite materials starts from the core of the mold. The degree of crystallinity is mentioned in Figure 5c and Table 2. The degree of crystallinity is directly proportional to the enhancement of mechanical properties of composite materials; see Figure 11, Figure 12, Figure 13, Figure 14 and Figure 15 and Table 3.

### 3.3. SEM Characterization of Composites

The representative SEM micrographs of PP-PCCF, PP-PCPESF, and PP-PET are presented in Figure 7, Figure 8, and Figure 9, respectively. Figure 7a shows a SEM image of pure PCCF. Microfibrils, porosity, distortion, and damage appear on the surface of fibers. These defects can contribute to lowering the quality and performance of recycled composite materials. The pure PP’s surface appeared smooth; see also Figure 6 and Figure 10. In any case, composites (with 10, 30, and 40% wt. fiber loadings) expressed the presence of PCCF, PCPESF, and PCPETF fibers. The existence of fibers produces surface defects like micro cracks and asperities. In pure PP, particles of equal size are compressed under high temperatures. PP powder’s particles of the same nature are cooled at the same rate to form an embryo. Hence, a PP polymer product with a uniform surface is formed (Figure 6 and Figure 7b). Figure 7c shows the asperities and evidence of PCCF fibers on the surface of PP-PCCF-10% wt. composite material. Micro-cracks and voids appeared on the surface (Figure 7d) of PP-PCCF-30% wt. composite material due to the PP-PCCF interface, poor adhesion, and the nature of cotton fiber. PCCF increment makes composites hard and brittle. Therefore, surface defects become prominent, see Figure 7e.

The addition of another material (for instance, PCPESF and PCPETF) as a reinforced phase changes the thermodynamic [126], chemical, and physical properties of composites [127]. Thermally, the synthesized composites melt, degrade, and recrystallize within a specific range of temperature [128]. The oriented and random incorporation of fiber phase (for instance, PCPESF, see Figure 8a) allows composites to cure at different cooling rates. The difference in temperature as a thermal gradient produces expansion or contraction in composite materials. Therefore, it appeared as micro pits on the surface of PP-PCPESF with 10% wt. composite material, see Figure 6 and Figure 8b. Figure 8c represents the appearance of PCPESF and matrix–fiber poor adhesion, grooves, and highly rough areas on the surface of PP-PCPESF-30% wt. recycled composite. The degree of the mentioned micro defects has become prominent on the surface of PP-PCPESF-40% wt. fabricated composite (Figure 8d) due to the highest amount of PCPESF and other parameters. The PCPETF in the form of flakes was used for the fabrication of PP-PCPETF composite materials. The PCPETF flakes exhibit surface deformation and rough regions (Figure 9a). The plastic deformation and distorted regions can cause poor adhesion between the PP and PCPETF interface. The micro pits (Figure 9b) become more evident due to the flake-like shape and size of PCPETF. Figure 9c has manifested some uniform regions on the surface due to crystallinity and mutual PP-PCPETF compound (composite) formation. However, conventional asperities and line cracks were observed on the surface of PP-PCPETF-40% wt. composite material, see Figure 9d [127].

### 3.4. Surface Roughness Evaluations of Composites

The quantitative surface analysis of recycled composite groups is shown in Figure 10. The minimum values of surface roughness parameters were found for pure PP (Figure 10a). Naturally, the increase in fiber addition (i.e., PCCF, PCPESF, and PCPETF) produced various surface defects (Figure 6, Figure 7, Figure 8 and Figure 9). Therefore, according to Figure 10a–c, the values of Ra, Rq, Rp, Rz, and Rt enhance individually for each composite material with an increase in fiber loading from 0 to 40%. All composite materials with 30% wt. fiber loading manifested complex behavior. The surface-roughness parameters of PP-PCPETF composite materials are greater than the PP-PCPESF and the PP-PP-PCCF composite family.

### 3.5. Mechanical Testing and SEM Characterization of Composites

#### 3.5.1. Tensile Testing and SEM Analysis of Fracture Surface

The results of tensile properties of PP-PCCF, PP-PCPETF, and PP-PCPES composites with different fiber loading are shown in Figure 11. The experimental data indicate that tensile strength (Figure 11a) and tensile strain (Figure 11c) decrease with an increase in PCCF, PCPETF, and PCPESF fiber contents. The increment of fiber loadings (all PCCF, PCPESF, and PCPETF) decrease the adhesion between fiber–matrix interfaces [129,130]. Additionally, the surface and cross-sectional defects can offer hurdles to transferring of load [131]; see Figure 6, Figure 7, Figure 8, Figure 9 and Figure 10 and Table 3. Therefore, these conditions decrease the performance and quality of developed composites [132]. On the other hand, the modulus of elasticity (Figure 11c) increases naturally with an increase in the amount of fiber in composite materials [133]. As fiber-reinforced composites are a combination of two unusual polymers, hence matrix and fiber phases help to resist environmental impacts and transfer of loads, respectively, see Figure 6. 

The ability of composites to withstand impact loads is improved with a rise in fiber amounts, see Table 3. The improvement in impact energy for PP-PCPETF composites is observed more compared to PP-PCPESF composites. 

According to dynamic mechanics, the stretching force propagates in composites through fibers from one matrix phase to another. The application of force appeared as deformation (change in length) in composite materials. Pure PP and composite materials (PP-PCCF, PP-PCPETF, and PP-PCPESF) consist of crystalline and amorphous phases, see Figure 6. The PP-PCCF, PP-PCPES, and PP-PCPET composites with 10% wt. show the highest level of crystallinity. Crystallinity is an indication of good adhesion between matrix and fiber phases. The crystalline phases resist the creation of deformation and ease the load-transferring phenomenon [134]. However, the increase in fiber content resists the transfer of load [135]. Therefore, all composites with 30 and 40% fiber loadings have the highest level of resistance toward load application. Additionally, the higher number of amorphous phases and lower level of crystallinity also enhanced brittleness and lowered ductility. Finally, the fracture appeared in the form of fibers pullout, see Figure 11d.

#### 3.5.2. Flexural Testing and SEM Analysis of Fracture Surface

The results of flexural properties of PP-PCCF, PP-PCPETF, and PP-PCPES composites with different loading fiber loading are shown in Figure 12. According to Figure 12a and Table 3, the PP-PCCF 40% wt. composite material expresses the highest value of flexural strength of 57 MPa. Similarly, pure PP, PP-PCPESF 40% wt. and PP-PCPETF 40% wt. have a value of flexural strength of 42 MPa, 41 MPa, and 39 MPa. The higher value of flexural strength of PP-PCCF 40% wt. may be due to the nature of PCCF and PP material and the direction of application of force. However, in comparison, the PP-PCCF 30% wt. shows the lowest value of flexural strength of 35 MPa. Furthermore, composite materials with 30% fiber loadings (PCCF, PCPESF, and PCPETF) have complexity in mechanical behavior. That is why the other two, the PP-PCPESF 30% wt. and PP-PCPETF 30% wt. composite materials have values of flexural strength of 43 MPa and 40 MPa, respectively. Besides this, the PP-PCCF 10% wt., PP-PCPESF 10% wt., and PP-PCPETF composite materials show reasonable values of flexural strength of 51 MPa, 41 MPa, and 40 MPa, respectively.

Figure 12b and Table 3 represent the values of the flexural constant of various manufactured composite materials; the value of the flexural constant increases with an increase in the amount of fiber loadings. The addition of reinforced materials (PCCF, PCPESF, and PCPETF) forms special compounds with the PP matrix [136]. The composite materials with 30% fiber loadings present peculiar behavior due to the transformation of ductile to brittle behavior. According to Figure 12c and Table 3, the ductility was found to be higher for PP-PCCF 10% wt. and PP-PCPESF 10% wt. composite materials due to flexural strain values of 14 and 13, respectively. All other composite materials (30 and 40% wt. fiber loadings) show brittle fracture. Moreover, tensile investigations do not support the ductile behavior of composite materials with 30 and 40% wt. fiber loadings, see Figure 11a–d and Table 3.

Figure 12d expresses the representative SEM image of flexural failure of PP-PCPESF 40% wt. composite material. In this case, the failure occurs in compressive conditions due to reversion of direction of force, plastic deformation, fiber pullout, and hence fracture of composite material. Initially, at and above the yield point, the deformation of reinforced fibers becomes permanent. After that, at a point of flexural strength (Figure 12a and Table 3), the composite materials withstand the highest compressive strength, and fiber pullout comes into existence. The flexural constant (Figure 12b) helps to evaluate the failure mechanism (ductile or brittle) of composite materials. Finally, the fracture of composite materials occurs at a specific point.

#### 3.5.3. Impact Testing and SEM Analysis of Fracture Surface

The results of the impact energy of all composite materials are shown in Figure 13a. The highest value was found for PP-PCCF 40% wt. composite material. In the comparison of the impact energy of developed composite materials, the PP-PCCF-10% wt., PP-PCCF 30% wt., and PP-PCCF 40% wt. composite materials show the highest values of impact energy of 4.8 kJ/m^2^, 4.4 kJ/m^2^, and 5.5 kJ/m^2^, respectively. However, the values of impact energy of all composite materials with PCPESF reinforcement and 10, 30, and 40% variations were 2.80 kJ/m^2^, 2.81 kJ/m^2^, and 3 kJ/m^2^, respectively. These values are lower than PCCF-reinforced-based composite materials, mainly due to the nature of fiber materials. The values of impact energy of PCPETF reinforced-based composite materials were intermediate between PCCF and PCPESF reinforced-based composites; see Figure 13a and Table 3. The sudden load transfer from fibers to the matrix phase causes fiber pullout, deformation, and fracture of the PP-PCPESF 40% wt. composite material collectively, as can be seen on the SEM micrograph, see Figure 13b.

### 3.6. Tribological Investigations and SEM Characterization of Composites

#### 3.6.1. Abrasion Testing and SEM Analysis of Abrasive Surfaces

The results of abrasive wear rates of PP-PCCF, PP-PCPESF, and PP-PCPETF composites are shown in Figure 14a and Table 4. Pure PP offers maximum resistance towards cutting and shearing (abrasive wear of PP is 3.09 × 10^−6^ mm^3^/Nm). Adhesion of PP particles, high rate of crystallinity (Figure 5c, Table 2, and Figure 6), and low surface roughness (Figure 7b and Figure 10a) also enhanced the tribological properties. The addition of fibers (PCCF, PCPESF, and PCPETF) produced micro pits, line defects, and microcracks (especially in PP-PCCF/ PP-PCPESF/PP-PCPETF with 10 wt.% composites); see Figure 7c, Figure 8b and Figure 9b. These defects provide stress concentration sites for deformation creation [137]. Therefore, it increases the value of abrasive wear rates. The values of wear rates of PP-PCCF, PP-PCPESF, and PP-PCPETF (with 10 wt.% fiber loading) composite materials were 3.54 × 10^−6^ mm^3^/Nm, 4.0 × 10^−6^ mm^3^/Nm, and 6.21 × 10^−6^ mm^3^/Nm, respectively. For further increase in the content of fibers, the surface defects (that appeared in PP-PCCF/PP-PCPESF/PP-PCPETF with 10 wt.% composites) transformed into surface asperities, high roughness areas and poor adhesion between matrix and fiber interface. The PP-PCPESF composites with 30 wt.% show abnormal behavior.

On the other hand, the behavior of PP-PCPETF composites with 30 wt.% is found in accordance with other values. The values of wear rates of PP-PCCF, PP-PCPESF, and PP-PCPETF (with 30% wt. fiber loadings) composite materials were 6.39 × 10^−6^ mm^3^/Nm, 3.54 × 10^−6^ mm^3^/Nm, and 6.40 × 10^−6^ mm^3^/Nm, respectively. The peculiar behavior of these composites is due to the transition from ductile to brittle behavior, see Figure 11, Figure 12 and Figure 13 and Table 3. At the extreme level of fiber addition (PP-PCCF/PP-PCPESF/PP-PCPETF with 40 wt.% fiber loadings), the micro defects (mentioned above) become prominent on the surface of composite materials, see Figure 6, Figure 7e, Figure 8d, Figure 9d and Figure 10. The composites are transformed into brittle materials permanently because of increases in strength, hardness, and other mechanical properties (Figure 11, Figure 12 and Figure 13 and Table 3). The wear rate values of such investigated PP-PCCF, PP-PCPESF, and PP-PCPETF composite materials with 40 wt.% fiber loadings were 6.39 × 10^−6^ mm^3^/Nm, 4.13 × 10^−6^ mm^3^/Nm, and 6.21 × 10^−6^ mm^3^/Nm, respectively, see Table 4.

The interaction between PP-PCCF/PC-PCPESF/PCPCPETF composites materials and SiC P150 grade sandpaper expressed a large variation in the values of coefficient of friction, see Figure 14b and Table 4. The average COF value of PP was 0.70. The applied normal load helps to produce adhesion between the polymer and counter-metallic surface. The hard particles of SiC material interact with a polymeric surface. The degree of adhesion relies on the apparent conditions of PP-PCCF/PP-PCPESF/PCPCPETF composite materials. During the mechanism, the applied load is transformed into energy dissipation and shear phenomena. Initially, the average COF values of PP-PCCF, PP-PCPESF, and PCPCPETF composite materials (with 10% wt. fiber addition of PCCF, PCPESF, and PCPETF) were increased due to asperities, line-like micro-cracks, stress concentration sites and poor adhesion at matrix–fiber interface. The shear between interacting surfaces causes elastic and plastic deformation. The highest value of COF (1.51) was observed for PP-PCCF composites with 40% wt. fiber loading. At the climax, the shear, tear (cutting), and plowing engender fracture of the composite. In SEM analysis (Figure 14c), the fracture of pure PP (reference material) appeared in the form of abrasive wear. Formally, during sliding, the moving composite pins encounter static hard particles of SiC sandpaper. Adhesion comes into existence at the composite pin surface-SiC hard particle interface. The surface defects (Figure 6, Figure 7, Figure 8, Figure 9 and Figure 10) act as a stress concentrator.

#### 3.6.2. Erosion Testing and SEM Analysis of Erosive Surfaces

The results of erosive wear rates of all recycled composite materials are shown in Figure 15 and Table 4. The minimum erosive wear rates of PP-PCPESF-10% wt. and PP-PCPETF-40% wt. were found to be 2 mm^3^/kg due to lower surface defects (Figure 7c and Figure 10a), and the nature of materials (PP and PCPETF), respectively. The erosive wear rate values of 7 mm^3^/kg and 8 mm^3^/kg belonged to PP-PCPESF-30% wt. and PP-PCPESF-40% wt., respectively. The minor increase in values was due to enhancement in fiber addition and surface defects. According to Figure 15a, the highest values were measured for the PP-PCCF group of composite materials. The PP-PCCF-10% wt., PP-PCCF-30% wt., and PP-PCCF-40% wt. composite materials corresponded to erosive wear rates of 19 mm^3^/kg, 15 mm^3^/kg, and 35 mm^3^/kg, respectively. Besides surface defects, the nature of sand particles and PCCF (lignin, hemicellulose, and microfibrils' individual effects) also have affected the COF values. However, the lowest values were observed for the PP-PCPETF group of composite materials. The PP-PCPETF-10% wt., PP-PCPETF-30% wt., and PP-PCPETF-40% wt. composite materials were related to wear rate values of 3 mm^3^/kg, 3 mm^3^/kg, and 2 mm^3^/kg, respectively. The lowering in erosive wear rate values can be expected due to PP-PCPETF interfacial adhesion, the nature of polymer materials, and PCPETF flake structure. Briefly, the lowest, intermediate, and highest values of erosive wear are associated with PP-PCPETF, PP-PCPESF, and PP-PCCF types of composite materials.

Figure 15b shows the representative SEM micrograph of PP-PCPESF-40% wt. In the erosion mechanism, initially, the impact collision of sand particles produced deformation on the surface of composite materials. The hard sand particles caused the shear and cutting of composite materials. Finally, cutting of composite material appeared as weight loss and erosive wear. The cutting of composite materials relies on physical parameters (like force, angle of cutting, speed, temperature, etc.), the nature of hard materials, soft materials, and other environmental functions.

### 3.7. Technical Aspects of Circularity and Commercial Applications

Circularity is still a theoretical concept in nature. In our previous research, Hussain et al. [138] presented paradigms and technical strategies for the implementation of circularity in polymer composite industries. The decrease in quality and performance of polymer waste occurs due to extensive use during service life. During service life, polymeric materials face numerous chemical and physical treatments. In chemical treatments and interactions, polymeric materials react with chemicals and cause corrosion, fatigue, and other phenomena. Similarly, continuous, and periodic mechanical interactions produce slip and shear on the surface of polymeric materials. The shear process initiates plastic deformation, surface damage, distortion, and even fracture [138]. At the end of the service life of polymeric products, the performance and quality decreased. Physical assessment, mechanical testing, tribological investigation, SEM evaluations, and surface roughness measurements can help in the selection of a suitable recycling technique. After selection and initial physical testing, the PC waste of natural and synthetic polymers is cut and ground into fine fibers. Fine fibers, as a reinforced phase, impart mechanical properties to composite materials. PP PCCF, PCPESF, and PCPETF are commercial polymer materials. Similarly, injection molding is also an industrial processing technique. Therefore, the manufacturing of PP-PCCF, PP-PCPESF, and PP-PCPETF smart composite materials can be considered commercial for various applications.

The performance and quality of recycled composite materials are tested using various analytical techniques. The DSC and TGA confirmed that all recycled composite materials could withstand room and optimum higher temperatures during service life, see Figure 5a–d, respectively. A single-factor ANOVA was also conducted regarding melting point, crystallization temperature, degree of crystallinity, and degradation temperature. The results are shown in Table 5. The *p*-value and F-value of all composite materials confirm thermal stability. According to Figure 10, micro defects exist mostly on composite materials with 30 and 40% fiber loadings. According to Table 5, the average surface roughness, especially Ra, was significant with an F-value and a *p*-value of 0.77 and 0.644 (Table 5), respectively. The critical suitability of composite materials for commercial applications is analyzed using tensile testing. PP-PCCF-10% wt. and PP-PCPESF-10% wt. exhibit good flexibility (Figure 11c) (in terms of strain and hence deformation), stiffness (elastic modulus), and with standability at higher loads, see Figure 11b,c. Such types of smart composite materials can have potential applications in the automotive, civil, aerospace, and nuclear industries. The PP-based heavy and low-weight composite materials are used for shielding from gamma rays in the energy range of 59.5–1332.5 keV. The pure PP and composite mate can be mixed with heavy metals, polymer virgin, and recyclable materials [139,140,141]. The ductile to brittle transition of developed composite materials appeared at 30% fiber loading. However, the modulus of elasticity increased, and tensile strength showed fluctuations in the mentioned values. Therefore, the PP-PCCF-30% wt., PP-PCPESF-30% wt., and PP-PCPETF-30% composite materials are only suitable for static loads and environmental impact applications. The brittleness has become constant for PP-PCCF-40% wt., PP-PCPESF-40% wt., and PP-PCPETF-40% wt. composite materials. Such hard and stiff composite materials can be utilized for insulation, tableware, marine boats, electrical fittings, domestic appliances, and other products. The tensile strength, elastic modulus, and tensile strain yield an effect size of 89.6%, 0.2%, and 4 × 10^−4^% with *p*-values of 0.896, 0.002, and 4 × 10^−6^, respectively. However, F-values of composite materials for tensile strength, elastic modulus, and tensile strain behavior were 0.50, 3.14, and 6.51, respectively [142,143].

Besides tensile, bend testing is also considered important for further investigations and confirmation of commercial applications of composite materials, see Figure 12 and Table 3. In the reverse application of load (compressive), the values of flexural strength of all composite materials were found to be higher than tensile strength. Similarly, the values of the flexural constant of all composite family materials were also higher than the tensile modulus of elasticity. Higher strength and flexural constant are an indication of thin restoration and stiffness in flexed conditions. It was also noted that flexural deformation (flexural strain) of polymer composite materials was also higher than that of tensile strain.

Additionally, flexural strength, constant, and strain yield an effect size of 86%, 5%, and 0.80% with *p*-values of 0.86, 0.05, and 0.008, respectively. Similarly, F values of flexural strength, constant and strain behavior were 0.51, 2.035, and 2.891, respectively (Table 5). The flexural tests also confirmed the potential applications of recycled composite materials.

Sometimes, composite materials also face impact loads during service life during static or dynamic conditions. Therefore, impact tests measure the impact energy of composite materials for such conditions, see Figure 13a,b and Table 3. The impact energy of composite materials creates an effect size of 60% with a *p*-value of 0.60. Moreover, the F-value of impact energy behavior was 0.823. The impact test and ANOVA statistical analysis justified the potential use of fabricated composite materials for various applications, see Table 5.

The surface performance and quality of composite materials are subjected to tribological tests. Mostly, abrasive wear occurs between manufactured composite materials and hard particles of silica sandpaper. Besides surface defects, all composite materials show good abrasion resistance values in the range of 3.09 × 10^−6^ mm^3^/Nm to 6.39 × 10^−6^ mm^3^/Nm, see Figure 14a and Table 4. Therefore, it can face environmental impacts during service life. The environmental impacts can also appear in the form of fatigue, creep, corrosion, or erosion mechanisms. The statistical analysis was also performed with a 13.75 sum of squares and 9 degrees of freedom, see Table 5. The abrasive wear of composite materials creates an effect of 98.7% with a *p*-value of 0.987. Furthermore, the F-value of the abrasive behavior of composite materials was 0.238. 

The interaction between the composite material and silica sandpaper also produces heat energy; see Figure 14b,c and Table 4. Amorphous surface, surface defects, and surface roughness reduce the adhesion between surfaces of composite materials and hard silica sand particles, see Figure 6, Figure 7, Figure 8, Figure 9 and Figure 10. Heat energy enhances the temperature of composite materials and softens them. The higher values of COF are an indication of the production of heat energy. However, lower values of COF are a sign of good adhesion between two interacting bodies during various motions. According to ANOVA (Table 5), the COF values of composite materials yield an effect of 91.4% under the influence of a *p*-value of 0.914. Similarly, the F-value of the COF behavior of composite materials was 0.428. The surface quality can be increased using binders, mixers, heat treatments, and surface finishing techniques.

The quality of recycled composite materials against environmental impacts like water, humidity, elevated temperature, chemicals, and mechanical stresses is tested using erosion tests, see Table 4 and Figure 15b. The value of erosive wear of recycled composite materials varies from 9 mm^3^/kg to 35 mm^3^/kg. The excellent resistance to environmental impacts is due to the PP matrix material. PP imparts good temperature resistance, resistance to humidity, and other chemicals. According to statistical analysis (Table 5), the erosive wear yields an effect of 1.07 × 10^−7^% with a *p*-value of 1.07 × 10^−9^. Moreover, the composite materials impart erosive behavior with an F-value of 11.80. The results of our fabricated composites were also compared with the outcomes of other reinforced virgin composite materials. A reasonable match was found regarding tensile, bending, impact, and other properties [144]. 

## 4. Conclusions

In this study, PP-PCCF, PP-PCPESF, and PP-PCPETF post-consumer fiber-reinforced composite materials were fabricated using injection molding with 0, 10, 30, and 40% fiber loadings. The developed composite materials were found to be thermally stable. Subsequently, the surface, mechanical, and tribological properties are as follows:

- All composite materials with 10% wt. fiber loadings exhibit lower surface roughness values, smooth surface, and minimum micro defects. However, voids, pits, microcracks, and rough areas appeared on the surface of PP-PCCF, PP-PCPESF, and PP-PCPETF composite materials with 30 and 40% wt. fiber loadings. Moreover, Ra, Rq, Rp, Rz, and Rt surface roughness parameters were also higher;
- The tensile, impact, and flexural properties of produced composites are linearly related to the nature, amount, and size of fibers. PP-PCPESF 10% wt. shows the highest values of tensile strength (29 MPa) and strain (10%) with the reasonable value of modulus of elasticity (1401 MPa). Similarly, tensile strength, strain, modulus of elasticity, flexural strength, strain, impact energy, and flexural constant of all other composite materials were found reasonable for potential commercial application;
- Collectively, composite materials with 10% wt. fiber loadings show suitability for structural applications due to good ductility, plastic deformation, stiffness, and standability at higher loads. At 30% wt. fiber loadings ductile to brittle transition occur due to the complex behavior of composite materials. However, composite materials with 40% fiber loadings exhibit suitability for environmental applications due to higher brittleness and stiffness, and impact energy;
- The values of abrasive wear with values in the range of 3 × 10^−6^ mm^3^/Nm to 6.5 × 10^−6^ mm^3^/Nm have manifested very good surface quality of fabricated composite materials. Similarly, manufactured composite materials can also withstand environmental impacts due to minimum values of erosive wear in the range of 2 mm^3^/kg to 35 mm^3^/kg;
- The statistical ANOVA predicts the potential use of recycled composite materials in various structural and environmental applications.

## Figures and Tables

**Table 1:** The average length, diameter, and area measurement of PCCF, PCPESF, and PCPETF ground fibers.

Post-Consumer Reinforced Fiber	Length (mm)	Diameter (µm)	Area (µm^2^)
PCCF	3.000 ± 0.001	18.000 ± 0.002	250.000 ± 0.004
PCPESF	3.500 ± 0.001	17.000 ± 0.002	245.000 ± 0.004
PCPETF	0.310 ± 0.0002	0.010 ± 0.002	0.190 ± 0.0004

**Table 2:** The demonstration of numerical values of melting, crystallization, degradation temperature, and degree of crystallinity of all developed composite materials.

Composite Family	Melting Temperature (°C)	Crystallization Temperature (°C)	Degradation Temperature (°C)	Degree of Crystallinity (%)
Pure PP	169 ± 2	118 ± 4	475 ± 5	42 ± 5
PP-PCCF 10% wt.	173 ± 2	105 ± 4	470 ± 5	44 ± 5
PP-PCCF 30% wt.	173 ± 2	108 ± 5	461 ± 6	33 ± 4
PP-PCCF 40% wt.	169 ± 1	118 ± 5	455 ± 7	24 ± 5
PP-PCPESF 10% wt.	171 ± 2	115 ± 3	455 ± 5	44 ± 3
PP-PCPESF 30% wt.	167 ± 2	120 ± 2	465 ± 4	38 ± 4
PP-PCPESF 40% wt.	167 ± 2	115 ± 3	470 ± 4	27 ± 4
PP-PCPETF 10% wt.	170 ± 1	117 ± 1	445 ± 7	39 ± 4
PP-PCPETF 30% wt.	169 ± 1	118 ± 1	460 ± 8	30 ± 3
PP-PCPETF 40% wt.	167 ± 1	117 ± 1	470 ± 8	30 ± 4

**Table 3:** The results of tensile, flexural, and impact properties of natural and synthetic-reinforced developed composite materials.

Composite	Tensile Testing			Bend Test			Impact Test
PCCF Content (% wt.)	Tensile Strength σ(MPa)	Tensile Strain (%)	Modulus of Elasticity E(MPa)	Flexural Strength σ(MPa)	Flexural Strain (%)	Flexural Constant E(MPa)	Impact Energy (kJ/m^2^)
PP	31 ± 2	10 ± 1.75	1226 ± 325	42 ± 6	14 ± 2	1172 ± 475	4.9 ± 0.30
PP-PCCF 10% wt.	26 ± 2	8 ± 1.50	1476 ± 400	51 ± 6	12 ± 2	2069 ± 475	4.8 ± 0.30
PP-PCCF 30% wt.	24 ± 2	4 ± 1.75	2521 ± 425	35 ± 6	10 ± 2	2130 ± 475	4.4 ± 0.30
PP-PCCF 40% wt.	22 ± 2	4 ± 1.75	2751 ± 430	57 ± 6	5 ± 2	3780 ± 475	5.5 ± 0.30
PP-PCPESF 10% wt.	29 ± 2	10 ± 2	1401 ± 125	41 ± 1	13 ± 1.25	1820 ± 325	2.80 ± 0.60
PP-PCPESF 30% wt.	25 ± 2	5 ± 2	1526 ± 125	43 ± 1	12 ± 1.25	2119 ± 325	2.81 ± 0.60
PP-PCPESF 40% wt.	23 ± 2	4 ± 2	1591 ± 125	41 ± 1	9 ± 1.25	2205 ± 325	3 ± 0.60
PP-PCPETF 10% wt.	25 ± 2	5 ± 2	1167 ± 85	40 ± 2	12 ± 1.50	1909 ± 275	2.60 ± 0.60
PP-PCPETF 30% wt.	22 ± 2	4 ± 2	1117 ± 85	40 ± 2	10 ± 1.50	2150 ± 275	3 ± 0.60
PP-PCPETF 40% wt.	22 ± 2	3 ± 2	790 ± 85	39 ± 2	9 ± 1.50	2140 ± 275	3.4 ± 0.60

**Table 4:** Results of abrasive wear, erosive wear, and COF of natural and synthetic reinforced composite materials.

Composite Family	Abrasive Wear (mm^3^/Nm)	Erosive Wear (mm^3^/kg)	COF
Pure PP	3.09 × 10^−6^ ± 0.10 × 10^−6^	9 ± 6	0.70 ± 0.15
PP-PCCF 10% wt.	3.54 × 10^−6^ ± 0.10 × 10^−6^	19 ± 7	1.11 ± 0.20
PP-PCCF 30% wt.	6.25 × 10^−6^ ± 0.10 × 10^−6^	15 ± 7	1.23 ± 0.20
PP-PCCF 40% wt.	6.39 × 10^−6^ ± 0.10 × 10^−6^	35 ± 7	1.51 ± 0.20
PP-PCPESF 10% wt.	4.03 × 10^−6^ ± 0.05 × 10^−6^	2 ± 2	1.05 ± 0.10
PP-PCPESF 30% wt.	3.54 × 10^−6^ ± 0.05 × 10^−6^	7 ± 2	1.05 ± 0.10
PP-PCPESF 40% wt.	4.13 × 10^−6^ ± 0.05 × 10^−6^	8 ± 2	1.17 ± 0.10
PP-PCPETF 10% wt.	6.21 × 10^−6^ ± 0.10 × 10^−6^	3 ± 2.5	1,12 ± 0.15
PP-PCPETF 30% wt.	6.40 × 10^−6^ ± 0.10 × 10^−6^	3 ± 2.5	1.25 ± 0.15
PP-PCPETF 40% wt.	6.21 × 10^−6^ ± 0.10 × 10^−6^	2 ± 2.5	1.14 ± 0.15

**Table 5:** Single-factor ANOVA test of thermal, surface roughness, tensile, bending, abrasion, and erosion properties of natural and synthetic reinforced composite materials.

Source of Variance	Composite Material’s Behavior	Sum of Square (SS)	Degree of Freedom (df)	F Value	*p*-Value
Between groups	Melting point	170.79	9	0.004	1
Between groups	Crystallization temperature	986.67	9	0.060	0.999
Between groups	Degree of crystallinity	205.37	9	1.298	0.262
Between groups	Degradation temperature	3057.08	9	0.01	1
Between groups	Arithmetic average surface roughness value	34.70	9	0.770	0.644
Between groups	Tensile strength	330.15	9	0.50	0.869
Between groups	Modulus of elasticity	14,203,574.67	9	3.410	0.002
Between groups	Tensile strain	276.95	9	6.51	0.000004
Between groups	Flexural strength	1251.4	9	0.51	0.860
Between groups	Flexural constant	15,374,265.15	9	2.035	0.054
Between groups	Flexural strain	226.04	9	2.891	0.008
Between groups	Impact energy	20.545	9	0.823	0.60
Between groups	Abrasive wear	13.75	9	0.238	0.987
Between groups	COF	17.318	9	0.428	0.914
Between groups	Erosive wear	3733.504	9	11.80	1.07 × 10^−9^

## Data Availability

The raw/processed data required to reproduce these findings cannot be shared at this time as the data also form part of an ongoing study.
